# Why the So-Called Gold Annealer Should Be Used in Giving the Heat Treatment to Gold Foil

**Published:** 1906-08-15

**Authors:** M. L. Ward

**Affiliations:** Ann Arbor, Mich.


### WHY THE SO-CALLED GOLD ANNEALER SHOULD BE USED IN GIVING THE HEAT TREATMENT TO GOLD FOIL.

BY M. L. WARD, D.D.S., ANN ARBOR, MICH.

Read before the Southwestern Michigan Dental Society, at Niles, Mich., April 10, 1906.

It is with reluctance that, in the midst of this porcelain epidemic, I attempt to present anything which will stimulate an interest in the subject of gold filling, which, in the minds of a great many, is being too rapidly consigned to obscurity.

It is getting more and more obvious every day that altogether too many operators who have served their patients well for years are failing to realize the intoxicating effect of "something new," and are being led astray by the porcelain enthusiasts who often cast aspersions and caustic remarks at the men who are delinquent in taking their porcelain in the usual large doses that are now being administered.

It is true that in some cases more artistic work can be done with porcelain, but who among us would enumerate the requisites for a good filling material and place color above durability when the operation was located in a remote part of the mouth? But the specialists say, "the convenience to both operator and patient in inserting the filling is a greater blessing than anything that was ever given to the profession before, and continue the tirade against some of our most conservative men, though they are undoubtedly the best ones, by terming them "partial converts."

It is certainly incomprehensible that men claiming to be leaders in this progressive period of dentistry can be (to put it mildly) so inconsistent as to visit some of the most energetic and conscientious men in the profession, and at the sight of a gold annealer, remark that it no longer has a place in their office, that the user was not converted; or that if he believed in porcelain at all he believed in it altogether, and that if porcelain was good for one place it was good for every place in the mouth.

I would affirm in the most positive manner that those who indulge in such remarks have not the remotest claim to the position they assume to occupy; nor are they accomplishing the good that they might, by teaching the principle that porcelain should be inserted in a most artistic manner in all visible locations, and only in such remote places as were not exposed to the forces of mastication.

I feel that we owe an ever-lasting debt of gratitude to the porcelain workers for the impetus they have given this subject, though I am confident that the near future will prove that porcelain, like all other filling materials, has its limitations, and that these same enthusiasts will be forced to admit that the field for porcelain fillings is not limited by the SKILL but by the JUDGMENT of the operator.

Is the gold annealer to be a thing of the past, as the porcelain enthusiasts say," or is its field of usefulness to be lessened by the judicious insertion of porcelain and gold inlays?

Has porcelain come to substitute any or all of our filling materials completely, is it only another one with which we can imitate nature more closely? These are the questions which confront us now and are being decided in favor of the latter by many men who are worthy to be classed with the broadest in the profession.

One might as well try to convince Dr. Honey that the gold inlay was not preferable to the porcelain in many locations, or to teach Dr. Hines that standard alloy is not preferable to the high percentage silver alloys in some cases, as to try to teach good operators the total extinction of the gold annealer and gold foil as a filling material.

It is probable the amount of gold foil used for fillings has been materially lessened by the introduction of porcelain and gold inlays, but it is by no means supplanted by either of them, and must be considered one of our standard filling materials for some time to come, at least. With the rapid advancement that has been made in dental electricity within the last few years, has come a device which has not claimed the attention that is due it as a most efficient means of preparing gold foil for filling.

This device, "the so-called gold annealer," a very misleading name, enables one to heat the gold sufficiently to effectively drive off all deposits from its surface without contaminating the gold. It is quite generally understood now that all gold foil for filling teeth, is and should be, as pure as it can be made, though it is claimed by many that its cohesiveness is not interferred with, when slightly alloyed with certain metals.

It is also well known that there is little difference between cohesive and non-cohesive gold, as it comes from the manufacturers, except that the non-cohesive has ammonia gas condensed on its surface.

They were both *purified*, *beaten* and *annealed* in the same manner but just previous to marketing, a part of the lot is passed through ammonia fumes and marked non-cohesive simply because these fumes form an inperceptible film which, while not affecting the purity of the gold itself, prevents intimate cohesion of the different layers of gold. From this we may see that a pellet of cohesive gold may be made non-cohesive by exposing it to the influence of ammonia gas and this pellet thus rendered non-cohesive may in turn be made cohesive by driving off the gas with heat. This latter process is what we actually accomplish in our every day operations with non-cohesive foil when we heat it and it is in no way intended to anneal the gold.

The "so-called annealing" of gold foil is not analogous to, nor in any way connected with, the annealing of plate gold which has become hard by hammering and has to be raised to a red heat to relieve it of the state of strain. Much of the difficulty experienced by operators in the insertion of gold is undoubtedly due to faulty methods of giving it the heat treatment, and even among operators who are sufficiently skilled to obtain good results by the ordinary methods, there is much to be gained by adopting some of the more recent advantages that are afforded us in the use of the "so-called electric gold annealer." Most operators are in the habit of heating their gold by passing it through the flame of a spirit lamp, or a Bunsen burner, but in either instance we are never certain of always having a pure flame; besides there is not one in a dozen that understands that he is removing gases and not developing cohesion, which is an inherent quality of the pure clean gold itself. To remove these gases uniformly and completely a comparatively low heat and more time is required and this is best accomplished with an electric annealer. It has been shown by experiment that the cohesive property of ordinary gold foil as it comes from the manufacturers shows itself at about 250 degrees F. and increases from this point to about 375 degrees F., after which nothing is gained. As this heat is not visible many dentists who have not tried the gold annealer with its low heat believe that gold annealed in this way is not thoroughly annealed, but clinical use shows it to be most cohesive.

It must not be understood that this amount of heat would be sufficient if the gold is held in the pliers where a large amount of heat is conducted away nor would it be sufficient if the gold were subjected to it for a short time only. It is, however, sufficient if the gold has not been too tightly rolled and the ends of the pellets are not crimped by cutting with the shears so that the gases cannot escape readily. I do not mean to infer that these gases cannot be driven off quite uniformly by passing the gold through the flame of a spirit lamp or a Bunsen burner, nor that for the general work of filling teeth it has not been done very well in the past; but who is there among us who cannot recall some nice operation that he has done that later on developed pits on its surface due to failure of some piece of gold to weld? Who is there that can doubt but that gold rolled by machinery with the ends of the pellets wide open, as they are furnished us now, when placed upon an electric annealer will develope a higher and more perfect cohesion than any gold that was ever rolled by hand and heated by holding it with the pliers in the different flames which are often vitiating and contaminating? The presence of moisture in the air always affects the alcohol flame, because of the great affinity which alcohol has for water. An undue humidity in the operating room often results in a vitiated flame which shows itself with a yellowish tinge. The flame from a Bunsen burner is more reliable than the alcohol flame, but it, too, will give a variety of results depending upon the location of the gold in the flame and the purity of the gas.

The lower part of such flames consist of gases, while the upper part is largely products of combustion, and when at their best it is very doubtful if gold coming in contact with them is not in danger of contamination. Granted that the flame used was in perfect condition, the other methods of procedure necessary to carry out this manner of annealing are certain to give unequal results. A pellet of gold that is picked up in the pliers and passed through the flame is quite certain to have from one-fourth to one-half of it between the plier points imperfectly annealed, even though the end of the pellet most remote from the pliers is heated to its melting point, and besides a portion of the pellet has been compressed so tightly that gases, no matter how easily driven off, would be mechanically held in the pellet, thus preventing a welding of the different layers. Some operators claim to obviate this difficulty by rolling the gold into a rope and then heat the entire rope on a sheet of mica.

This eliminates the danger of contaminating the gold with the gases and products of combustion, but is usually accomplished in so short a time that the gases cannot escape from a rope of gold unless the rope has been made very loose and the temperature has been previously high.

Should the operator succeed in removing the gases quite completely there is another objection, while it may appear a trivial one to some, it should be considered in this connection, viz., the impact of the scissors in cutting pellets from a rope of annealed gold compresses the ends so much that they are not obedient to the plugger point nor readily adapted to difficult angles and margins.

One operator claims to change ends with every pellet of gold, thus getting both ends of the pellet well heated, but even then he has not overcome the objection of compressing the pellet so that the condensed gases are mechanically held in the pellet.

Another operator claims to hold his gold in the flame with a plugger point and in this way avoid compressing the ends of the pellet and at the same time has a minimum amount of heat conducted away. While this method is better than the pliers, unless the plugger point is heated sufficiently to ruin its quality as a plugger, the gold is never perfectly annealed in the region of the point, besides there is a great liability of the products of repeated oxidation of the plugger point being incorporated in the filling only to be disclosed upon some conspicuous surface after some little attrition on the filling.

From what has been said it would seem that even the most casual observer should be convinced that, while beautiful gold fillings are being done without the electric annealer, it possesses fewer disadvantages than any other device that has been brought to our attention, and that gold that has been heated on it has a working quality that cannot be obtained in any other way.

The time saved by using the annealer instead of holding the gold in the flame is not a minor consideration, since all operations with gold foil consume a comparatively large amount of time. Even with operators who are rapid workmen it will be found to save them upward of 25 percent of the time ordinarily required to insert such fillings. Those who are not located where the electric current is at their command, should find the annealers that are made for gas and alcohol flames preferable to the naked flame, though if they are not *carefully managed*, they will not give much more uniform results than the flame itself, because of the uneven distribution of the heat over the annealing tray. In conclusion, I will say that I firmly believe that those who once become accustomed to the soft, though highly cohesive, property of gold prepared by machinery and heated on an electric annealer, will have no desire whatever to return to the older methods.

I also believe that the average operator, even though he sacrifices something on density and tensile strength, will, with this method, secure an adaptation that will raise the percentage of his successes.

### DISCUSSION.

W. E. HARPER, Chicago: From the several standpoints that were brought out in the paper, the minimum of time is required, and the better quality of the filling obtained by the use of the electric or some equivalent annealer. We rarely see fillings of six, eight and ten years' standing, without finding on their surfaces some pits or some indentations most often caused by faulty annealing. I have used the stand and tray with the alcohol lamp, and I accomplish what appears to be equivalent results both in the minimum of time and the conditions of service after years of wear. I don't imply that I am above making a mistake, or never find a filling of mine with a pit, but the general average of work should be much better with the electric annealer. I did, for some years, use the alcohol flame for lack of any better.

You can find on the market non-cohesive gold that cannot be made cohesive by any amount of annealing. In that case it is rendered non-cohesive by some other agent than ammonia gas. Gold that cannot be made cohesive is treated with something that cannot be driven off with heat. You cannot anneal gold with safety by carrying it through the flame with a pair of pliers unless you consume a large amount of time in so doing. It will be necessary, to properly anneal a piece of gold, to take hold of it twice, to put it down on the table and take it up again by the other end, otherwise you will certainly have a piece of gold in which you have highly developed cohesion in one part and imperfect cohesion in another part, which must result in an imperfect filling. In the ordinary distal or mesial filling, one that doesn't involve an angle, if the pellet is used, I would probably use what is called the one-half pellet. In a simple mesial cavity we use say fifty pieces of gold. My tray contains about forty-seven spaces in which gold is placed before beginning. Now just imagine the time that is lost, to say nothing of the quality of the annealing, if you spend five or ten seconds in the annealing by hand through the flame, it will easily amount to 25 percent of the time required to do the operation complete with the electric annealer. With the annealer you get a perfectly cohesive piece of gold, if it can possibly be made so by the application of heat. The saving of time and quality of filling is something that ought to appeal, I am sure, to every operator.

I am going to let the other fellow do the porcelain work, but I hold the porcelain work in very high esteem. It requires experience and skill, that few of us possess, and it requires a man do do a great deal of that work to do it well. I draw my conclusions from the work it has been my privilege to examine. I feel that the dentist who is doing the porcelain work instead of the gold work is not very apt to do it well unless he is getting a much better fee for his porcelain than for his gold fillings. A general complaint of dentists is that they can't get adequate fees for gold fillings. I don't believe that a man can do porcelain work unless he can get a better fee. I am still of the conviction that we can put in gold fillings in many instances much cheaper and much better than the porcelain, so I am one of the few that are sticking to gold.

DR. O. E. LANPHEAR: I have been urging the porcelain idea in my town. I think the trouble with the porcelain, for the man in the small town, is that he cannot get enough of the work to do, and cannot get the right fee, so he has got to depend on gold filling, and the annealer question with me is a rather hard one, because I havn't the facilities to use the electric annealer, and I find a good many other practitioners near by who are compelled to use the alochol flame. I think if we could have the ideal conditions, we would all go over to the electric annealer.

DR. H. A. SPRAGUE: In offices in which the alcohol flame is to be used, if gold is kept in ammonia fumes, and thus kept from contamination with other gases (an ammonia salt is formed, and that ammonia salt is the most volatile of all common salts), the gold is more easily annealed. The salts or gases that are on the so-called non-cohesive golds are not as easily driven off.

DR. C. H. WORBOYS: This electric gold annealer is a nice thing. I haven't bought one yet. This subject of annealing gold is a pretty hard one. It is older than I am in the business and I have seen some fillings that have stood mastication that were made with cohesive gold before anything better than alcohol flame or Bunsen flame was used. They have stood the work, though some have pits. I don't believe one of these pits is due to non-cohesive gold. If anything, it was the operator's failure to beat that gold down where it belonged. I believe that cohesive gold should be thoroughly malleted, and if it is not thoroughly malleted, the filling will have pits. So much depends on the operator doing his work well. You can use an electric annealer or an alcohol flame annealer, or gas flame annealer, but you want a clean flame. I presume that the annealer that will anneal sufficient gold for most small fillings at once is an advantage for time. It is a time saver. It is a good thing for the dentist and a good thing for the patient, but I don't want any patient to go away with the idea that the annealer does the whole business. If you use any annealer you will have to put the gold in place and plug the tooth and make a watertight filling. When you make a porcelain filling you have got to make a watertight cork also.

DR. HADLEY: I would like to say a word to substantiate the last speaker. I saw a patient recently who had six gold fillings put in twenty years ago and there is not a pit in one of them. They are as smooth as when they were put in, and there are no signs of decay. They were put in by a dentist of Coldwater.

DR. RUNYAN: I haven't an electric annealer, and my fillings have a good many pits in them, I think it may be because of this fact.

DR. C. H. LAND: I almost abandoned the inlay eighteen years ago, although I invented it. I abandoned it not because I thought it would not save the teeth, but because it showed a partial disfiguration in the joint that was visible, so that the majority of my patients would come back in six or eight months and ask me to remove the inlay because of the dark joint. I have inlays that are in good order, with the exception of the joint. The enamel is not the weak point. We should remember that every gold or porcelain crown and every bit of bridge work probably has worse joints than the inlay, but in the former they are out of sight and do no particular harm. To think that we must abandon gold is a mistake. A man cannot abandon amalgam, he cannot abandon gutta-percha, he cannot abandon gold. All of these things are useful, and will always have a place. So far as the alcohol flame is concerned, there is nothing about it that injures gold. It is only a question of how one anneals and for what purpose. There is no gas that escapes that would be injurious to gold. The gas stays on the surface, As for myself, I would use an electrical annealer because it is a nice thing. I can use gold and make it last as well as any one. It is all a question of locality and of what is best for the patient. As for porcelain, it is strictly the esthetic. Outside of that there are plenty of fillings equivalent to it. It remains for the specialist to carry out porcelain in the large cities. In the smaller towns one can use porcelain work only in a limited degree.

DR. WARD, closing discussion: Any gas will be condensed on the surface and is injurious. I don't mean to infer at all that good gold fillings haven't been put in before the electric annealer was known. But there are advantages in the use of the electric annealer, the saving of time being the principal one, and next the quality of gold is much superior, but you all know that a good filling does not need to be made of gold annealed by electricity.

